# Longitudinal GGT trajectories identify prognostic phenotypes in paediatric primary sclerosing cholangitis

**DOI:** 10.1016/j.jhepr.2026.101940

**Published:** 2026-06-26

**Authors:** Daniela Denier, Yiming (Emmett) Peng, Mansi Amin, Madeleine Aumar, Marcus Auth, Eleonora Druve Tavares Fagundes, Wael El-Matary, Alexandre R. Ferreira, Katryn N. Furuya, Nitika Gupta, Stephen Guthery, Simon Horslen, M. Kyle Jensen, Binita M. Kamath, Bart G.P. Koot, Kathleen M. Loomes, Cara Mack, Mercedes Martinez, Nadia Ovchinsky, Alexandra Papadopoulou, Emily R. Perito, Pushpa Sathya, James P. Stevens, M. Elizabeth Tessier, Pamela Valentino, Mark Deneau, Kuan Liu, Amanda Ricciuto

**Affiliations:** 1 Division of Paediatric Gastroenterology, Hepatology and Nutrition, Department of Paediatrics, Inselspital, Bern University Hospital, University of Bern, Bern, Switzerland; 2 Division of Biostatistics, Dalla Lana School of Public Health, University of Toronto, Toronto, ON, Canada; 3 Division of Gastroenterology, Hepatology and Nutrition, The Hospital for Sick Children, Toronto, ON, Canada; 4 Department of Pediatrics, Duke University School of Medicine, Durham, NC, USA; 5 Department of Pediatric Gastroenterology Hepatology and Nutrition CHU Lille, Univ. Lille, Inserm U1286 - INFINITE - Institute for Translational Research in Inflammation, Lille, France; 6 Alder Hey Children's NHS Foundation Trust, Liverpool, UK; 7 Faculty of Medicine of Federal University of Minas Gerais (UFMG), Hospital das Clinicas of UFMG, Belo Horizonte, Brazil; 8 Children's Hospital Research Institute of Manitoba, Department of Pediatrics, University of Manitoba, Winnipeg, MB, Canada; 9 Division of Gastroenterology, Hepatology and Nutrition, Department of Pediatrics, University of Wisconsin-Madison School of Medicine and Public Health, Madison, WI, USA; 10 Department of Pediatrics, Division of Gastroenterology, Hepatology and Nutrition, Children's Healthcare of Atlanta, Emory University School of Medicine, Atlanta, GA, USA; 11 Department of Pediatrics, Division of Gastroenterology, Hepatology and Nutrition, Intermountain Primary Children's Hospital Salt Lake City, University of Utah, Salt Lake City, UT, USA; 12 Department of Pediatrics, Division of Gastroenterology, Hepatology and Nutrition, Children's Hospital of Pittsburgh, University of Pittsburgh Medical Center, Pittsburgh, PA, USA; 13 Division of Gastroenterology, Hepatology and Nutrition, The Children's Hospital of Philadelphia and the University of Pennsylvania Perelman School of Medicine, Philadelphia, PA, USA; 14 Department of Pediatric Gastroenterology and Nutrition, Amsterdam Pediatric Abdominal Center, Emma Children's Hospital, Amsterdam UMC location University of Amsterdam, Amsterdam, The Netherlands; 15 Department of Pediatric Gastroenterology, Hepatology and Nutrition, Medical College of Wisconsin and Children's Wisconsin, Milwaukee, WI, USA; 16 Department of Pediatrics, Columbia University Irving Medical Center, New York, NY, USA; 17 Division of Pediatric Gastroenterology, Hepatology and Nutrition, Hassenfeld Children's Hospital, New York University Grossman School of Medicine, New York, NY, USA; 18 First Department of Paediatrics, University of Athens, Agia Sophia Children's Hospital, Athens, Greece; 19 Department of Gastroenterology and Hepatology, University of California San Francisco, San Francisco, CA, USA; 20 Department of Pediatrics, Division of Pediatric Gastroenterology, Hepatology and Nutrition, Memorial University of Newfoundland and Labrador, Janeway Children's Health Centre, St. John's, Newfoundland, Canada; 21 Pediatric Gastroenterology, Hepatology and Nutrition, Baylor College of Medicine, Texas Children’s Hospital, Houston, TX, USA; 22 Department of Pediatrics, Gastroenterology and Hepatology Division, University of Washington and Seattle Children's Hospital, Seattle, WA, USA; 23 Institute of Health Policy, Management and Evaluation, Dalla Lana School of Public Health, University of Toronto, Toronto, Ontario, Canada; 24 Department of Paediatrics, University of Toronto, Toronto, ON, Canada

**Keywords:** Cholestasis, Natural history, Disease progression, Risk stratification, Surrogate endpoint

## Abstract

**Background & Aims:**

Primary sclerosing cholangitis (PSC) is characterised by substantial clinical heterogeneity. Although normal gamma-glutamyl transferase (GGT) at 1 yr predicts favourable outcomes, the heterogeneous trajectories underlying this remain uncharacterised. We applied latent class mixed modelling to identify distinct GGT trajectory phenotypes in paediatric PSC and evaluate their prognostic significance.

**Methods:**

We analysed 782 children with PSC from the Pediatric PSC Consortium who had baseline GGT and at least one additional measurement over 3 yr. Latent class mixed models were fit to log-transformed GGT values, with optimal model selection based on Bayesian Information Criterion and entropy. Cox proportional hazards models assessed associations between trajectory clusters and transplant-free survival, adjusting for baseline characteristics. We used multinomial logistic regression to assess whether cluster membership could be predicted using early markers.

**Results:**

Four trajectory clusters were identified: persistently elevated (23%), moderate-stable (30%), elevated-declining (26%), and persistently low (21%). The 5-yr transplant-free survival differed markedly: 74%, 86%, 98%, and 96%, respectively (*p* <0.0001). The elevated-declining and moderate-stable clusters both had elevated baseline GGT but divergent outcomes, with the elevated-declining cluster achieving normalisation by Year 1. In adjusted analyses, persistently elevated and moderate-stable clusters had significantly higher transplant hazard compared with persistently low cluster (HR 6.45, 95% CI 2.87–14.5; HR 3.00, 95% CI 1.31–6.90). Transplant-free survival in the elevated-declining cluster was similar to that in the persistently low cluster (*p* = 0.22). Favourable clusters had younger age with higher prevalence of inflammatory bowel disease. Cluster membership could be predicted using baseline and 6-month GGT values (AUC 0.83).

**Conclusions:**

GGT trajectory phenotypes identify clinically meaningful PSC subgroups with distinct prognoses. Early biochemical response, rather than baseline values alone, predicts long-term outcomes.

**Impact and implications:**

Prognostic assessment in PSC has relied on cross-sectional biomarker measurements at single time points, limiting the ability to capture dynamic changes over time. By applying trajectory modelling to repeated GGT measurements in 782 children with PSC, we identified four distinct groups with significantly different transplant-free survival. Two clusters with similarly elevated baseline GGT followed divergent trajectories and had very different outcomes, information that cross-sectional assessment would miss. These trajectory phenotypes can be predicted using baseline and 6-month GGT values, offering clinicians an early tool to identify high-risk patients. Trajectory-based phenotyping may also facilitate clinical trial design by enabling patient enrichment strategies and providing an early surrogate endpoint.

## Highlights

• Latent class mixed modelling of serial GGT in 782 children with primary sclerosing cholangitis identified four distinct trajectory phenotypes.
• The phenotypes comprised persistently elevated, moderate-stable, elevated-declining, and persistently low GGT clusters.
• The 5-yr transplant-free survival varied markedly across phenotypes, ranging from 74% to 98% (*p* <0.0001).
• Children with persistently low GGT and those whose elevated GGT normalised had similarly favourable outcomes.
• Cluster membership was predictable from baseline and 6-month GGT (AUC 0.83), supporting early risk stratification.

## Graphical abstract

**Figure:**
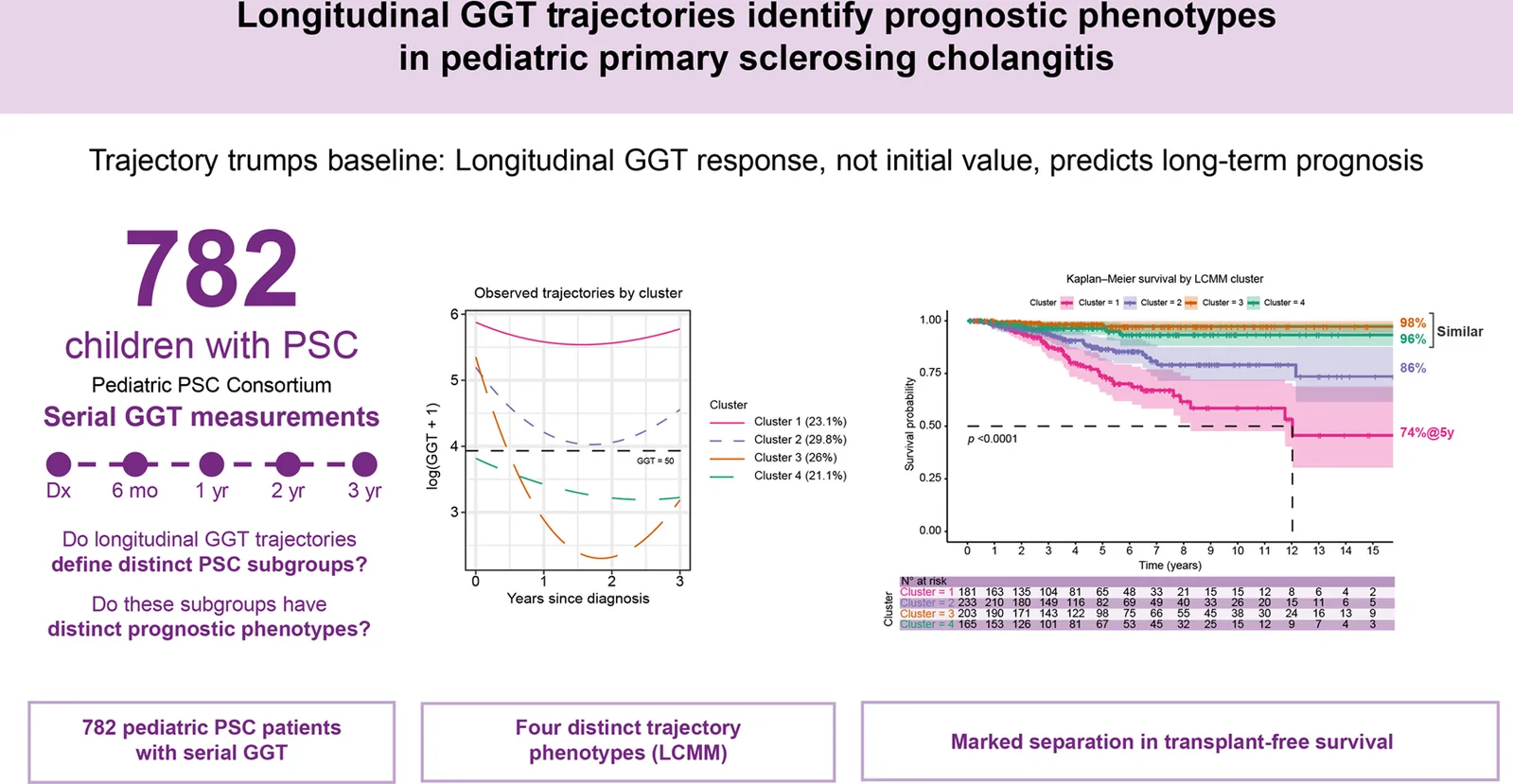

## Introduction

Primary sclerosing cholangitis (PSC) is a chronic cholestatic liver disease characterised by progressive bile duct inflammation and fibrosis, which can ultimately lead to cirrhosis, liver failure, and cholangiocarcinoma.^1^ PSC demonstrates substantial heterogeneity in clinical presentation, disease progression, and outcomes in paediatric and adult populations.^2^^,^^3^ This wide variability in disease course represents a fundamental challenge to precision medicine and clinical trials, as current tools inadequately distinguish patients destined for stable disease from those at risk for rapid progression requiring liver transplantation.

Gamma-glutamyl transferase (GGT) has emerged as a key prognostic biomarker in paediatric PSC. In a multicentre study, GGT normalisation or greater than 75% reduction within 1 yr of diagnosis predicted 5-yr event-free survival of 88–91% compared with only 61–67% in patients without such responses.^4^ These findings established GGT as a potential surrogate endpoint. GGT was subsequently incorporated into the Sclerosing Cholangitis Outcomes in Pediatrics (SCOPE) index, the first paediatric-specific PSC prognostic tool.^5^ However, studies have primarily examined GGT in a binary fashion (normalised *vs.* not) at discrete time points, potentially missing important information contained in the longitudinal patterns of GGT evolution over time. Similarly, adult studies have primarily examined other biomarkers, such as alkaline phosphatase (ALP), in a cross-sectional manner.

Trajectory modelling and clustering methodologies, including latent class mixed modelling (LCMM),^6^ have successfully identified clinically meaningful patient subgroups in diverse chronic liver and inflammatory diseases, such as chronic hepatitis B and Crohn's disease.^7^^,^^8^

Despite these advances in other diseases and the established prognostic importance of GGT in PSC, no studies have systematically characterised distinct longitudinal GGT trajectory patterns in PSC or examined whether such trajectories identify clinically relevant subgroups. We hypothesised that LCMM applied to serial GGT measurements would identify distinct trajectory clusters with differential baseline characteristics and clinical outcomes. In this study, we aim at (1) identifying distinct longitudinal GGT trajectory groups in a cohort of children with PSC, (2) determining the association between trajectory group membership and transplant-free survival, and (3) developing predictive models for cluster membership.

## Patients and methods

### Study design and setting

The Pediatric PSC Consortium is a retrospective, international, multicentre cohort comprising institutions across Europe, North and South America, the Middle East, and Asia. Standardised data were collected for each patient, including demographics, disease characteristics, medication exposure, and laboratory parameters at diagnosis, 6 months, 1 yr, and annually thereafter until 10 yr or transition to adult care. Data were abstracted and de-identified at local sites prior to central database entry. PSC diagnosis required a cholestatic biochemical profile, together with characteristic imaging or histopathology. Detailed methods have been described previously.^2^

### Study population

Children with PSC were eligible if they had baseline GGT (at PSC diagnosis) and at least one follow-up GGT measurement within 3 yr of diagnosis. We restricted analysis to laboratory values collected up to 3 yr from PSC diagnosis owing to substantial data attrition at later time points as a result of transition to adult care occurring at most centres around 18 yr of age. Moreover, we deemed early trajectory patterns most clinically relevant for prognostication. Patients were censored at liver transplantation and transition to adult care.

### Variables

We assessed patient demographics (sex, age at diagnosis), disease characteristics (large *vs*. small duct involvement,^9^ PSC with autoimmune hepatitis [AIH] features,^10^ concomitant inflammatory bowel disease [IBD]), and medication exposure (ursodeoxycholic acid [UDCA], corticosteroids, oral vancomycin for a PSC or IBD indication). Contributing investigators recorded AIH features based on standard diagnostic criteria, as previously reported.^2^ Medication exposure was treated as binary (ever exposed *vs.* never exposed).

### Longitudinal biomarkers

We analysed GGT values from diagnosis, 6 months, and 1, 2, and 3 yr. Given the high frequency of AIH features in paediatric PSC, we also examined alanine aminotransferase (ALT) at these time points to determine whether its inclusion in LCMM enhanced clustering or predictive performance.

### Outcome

The primary outcome was time to liver transplantation. We assessed the predictive utility of trajectory cluster membership against this outcome.

### Statistical methods

#### Latent class mixed modelling

We fit LCMM to repeated liver enzymes measurements to identify trajectory subgroups. Candidate model specifications varied by: (1) number of classes (K = 2, 3, or 4); (2) time trend (linear, quadratic, or natural cubic splines); (3) outcome scale (raw *vs.* log-transformed GGT); and (4) link function (linear *vs.* four-quantile splines). For each specification, initial values were obtained from the corresponding single-class model, with multiple random starts to reduce local-maximum risk.

Model selection was based on Bayesian Information Criterion (BIC), classification quality (entropy >0.6 indicating acceptable separation), and clinical interpretability. We modelled GGT alone, and also assessed ALT alone and combined GGT/ALT models.

#### Survival analysis

After determining the optimal LCMM, we compared patient characteristics across clusters and examined associations between cluster membership and transplant-free survival using Kaplan-Meier analysis and Cox proportional hazards regression. The multivariable model included covariates selected *a priori* based on clinical relevance: age, sex, large *vs.* small duct involvement, PSC with AIH features, concomitant IBD (present *vs*. absent), and baseline medications (UDCA, vancomycin, corticosteroids). Results are expressed as hazard ratios (HR) with 95% CIs. The proportional hazards assumption was verified by examining the interaction between covariates and time based on scaled Schoenfeld residuals. To explore the potential effect of classification uncertainty, we performed a sensitivity analysis restricted to patients with high posterior class membership probabilities >0.8 (patients with high certainty of cluster assignment).

#### Prediction of cluster membership

To assess whether trajectory cluster membership could be predicted early in the disease course, we developed multinomial logistic regression models using baseline and 6-month GGT values (log-transformed), with and without additional clinical covariates. Data were randomly split into 70% training and 30% test sets. Model performance was evaluated on the held-out test set using multiclass AUC and per-class AUCs.

#### Missing data

LCMM conditionally handles missing data under the missing-at-random assumption using maximum likelihood estimation, utilising all available data rather than imputing or discarding missing values. For the Cox regression, missing medication data without a documented start date were imputed as 'unexposed' in the primary analysis. We performed a sensitivity analysis using complete-case deletion to assess robustness. Missing data for other covariates (*e.g*. large *vs.* small duct) were treated as missing in all analyses.

### Statistical analysis

We summarised categorical variables as frequency (%) and compared them using the Chi-square test. We summarised continuous variables as mean (SD) or median (IQR), and compared them using *t* test or Mann-Whitney *U* test (two-group comparisons) or ANOVA or Kruskal-Wallis test (four-group comparisons), depending on distribution. Statistical significance was set at *p* <0.05 (two-sided). We also used standardised mean differences to contrast included and excluded patients to assess for selection bias. Analyses were performed using R version 4.4 with lcmm, survival, and glmnet packages (random seed 2025). Software details are provided in the Supplementary CTAT Table (R version 4.4.2 [R Foundation for Statistical Computing, Vienna, Austria]). No formal sample size calculation was performed, as all eligible patients meeting the inclusion criteria were analysed.

### Ethics

The institutional review board of each participating centre approved all research. This research was conducted in accordance with the Declaration of Helsinki guidelines of good practice. The need for informed consent was waived by each participating centre’s institutional review board given the retrospective nature of the study.

## Results

### Study cohort and patient selection

**Fig. 1:**
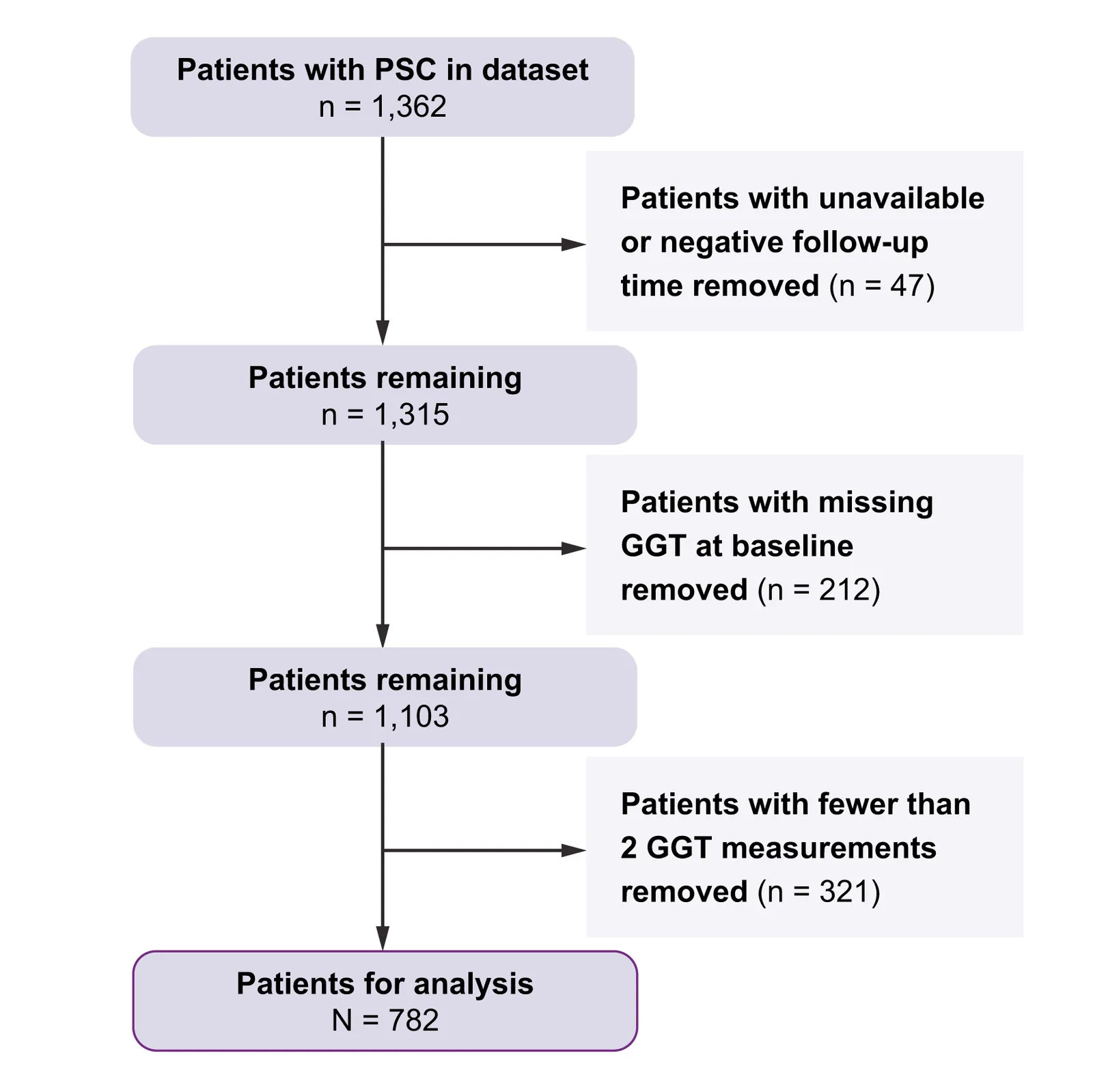
**Patient selection flowchart.** Flowchart depicting derivation of the analytic cohort from the PSC Consortium database. From 1,362 paediatric patients with PSC, 782 (57%) met inclusion criteria requiring baseline GGT measurement and at least one additional measurement over follow-up. Sequential exclusions: patients with missing or negative follow-up time (n = 47), missing baseline GGT (n = 212), and fewer than two GGT measurements (n = 321). GGT, GGT, gamma-glutamyl transferase; PSC, primary sclerosing cholangitis.

From an initial cohort of 1,362 paediatric PSC patients in the PSC Consortium database, 782 patients met inclusion criteria for trajectory analysis (Fig. 1). Patients were excluded if they had missing or negative follow-up time (n = 47), lacked baseline GGT measurement (n = 212), or had fewer than two GGT measurements in total (n = 321).

Compared with excluded patients, included patients had slightly older age at diagnosis and higher prevalence of PSC with AIH overlap features (Table 1). Included patients were more likely to have received UDCA and vancomycin at baseline. Baseline GGT levels were similar between groups.

**Table 1:** Characteristics of included and excluded patients.

Characteristic	Included (N = 782)	Excluded (n = 580)	*p* value	SMD
Demographics				
Age at diagnosis (yr), median (IQR)	13.0 (9.5–15.7)	12.3 (8.4–15.0)	0.004	0.145
Male sex, n (%)	476 (60.9)	345 (60.0)	0.789	0.018
Clinical features				
IBD present, n (%)	610 (78.0)	451 (77.8)	0.966	0.006
PSC with AIH features, n (%)	288 (36.8)	150 (27.7)	0.001	0.197
Large duct involvement, n (%)	684 (88.4)	493 (87.4)	0.654	0.029
Medications at baseline				
Ursodeoxycholic acid, n (%)	520 (79.3)	148 (65.8)	<0.001	0.306
Corticosteroids, n (%)	264 (45.6)	62 (39.0)	0.163	0.134
Vancomycin, n (%)	122 (21.5)	16 (11.1)	0.007	0.284
Laboratory values at baseline				
GGT (U/L), median (IQR)	212 (96–401)	185 (97–370)	0.521	0.089
ALT (U/L), median (IQR)	120 (54–227)	106 (52–205)	0.496	0.041
Haemoglobin (g/dl), mean (SD)	12.2 (2.1)	12.1 (2.1)	0.359	0.061
Platelets (×10^3^/μl), mean (SD)	325 (149)	318 (163)	0.496	0.045
INR, median (IQR)	1.1 (1.0–1.1)	1.1 (1.0–1.2)	0.002	0.124
Total protein (g/dl), mean (SD)	7.82 (0.86)	7.72 (0.99)	0.147	0.106
Albumin (g/dl), mean (SD)	4.0 (0.6)	3.9 (0.6)	0.019	0.149
Total bilirubin (mg/dl), median (IQR)	0.6 (0.4–1.1)	0.7 (0.4–1.6)	0.007	0.002
AST (U/L), median (IQR)	90 (45–177)	83 (43–154)	0.432	0.063
ALP (U/L), median (IQR)	339 (204–536)	394 (228–734)	<0.001	0.325
CRP (mg/dl), median (IQR)	0.65 (0.30–1.70)	0.70 (0.25–1.45)	0.949	0.044
ESR (mm/h), median (IQR)	29 (12–57)	31 (14–55)	0.829	0.042
IgG (mg/dl), median (IQR)	1,500 (1,052–2,023)	1,770 (1,390–2,310)	<0.001	0.486
Outcomes				
Liver transplantation, n (%)	82 (10.5)	87 (15.0)	0.016	0.136

Note: *p* values from Chi-square tests (categorical variables), *t* tests (continuous normally distributed variables), or Mann-Whitney *U* tests (continuous not normally distributed variables). AIH, autoimmune hepatitis; ALP, alkaline phosphatase; ALT, alanine aminotransferase; AST, aspartate aminotransferase; CRP, C-reactive protein; ESR, erythrocyte sedimentation rate; GGT, gamma-glutamyl transferase; IBD, inflammatory bowel disease; INR, international normalised ratio; PSC, primary sclerosing cholangitis; SMD, standardised mean difference.

The final analytic cohort of 782 patients had a median follow-up of 4.12 (IQR 4.75) yr. Among patients who started UDCA, 20% had a documented stop date over follow-up, and the median time on UDCA before stopping was 2.67 (IQR 1.02–4.97) yr. Only 4.7% stopped within 12 months of initiation. During follow-up, 82 patients (10.5%) experienced the outcome of liver transplantation. GGT measurements were available for all patients at baseline, 58% at 6 months, 81% at 1 yr, 54% at 2 yr, and 38% at 3 yr (Fig. S1). Fig. S2 shows GGT and ALT over time, highlighting the marked inter-individual variation. The overall trend lines (red) show modest average decline for both markers, masking the underlying clinically important heterogeneity.

### Identification of GGT trajectory patterns

We evaluated 36 candidate latent class mixed models. Models using log-transformed GGT exhibited substantially better fit compared with raw GGT models (Table S1). Among log-transformed models, those incorporating spline-based trajectory shapes provided superior fit to linear or quadratic specifications.

**Fig. 2:**
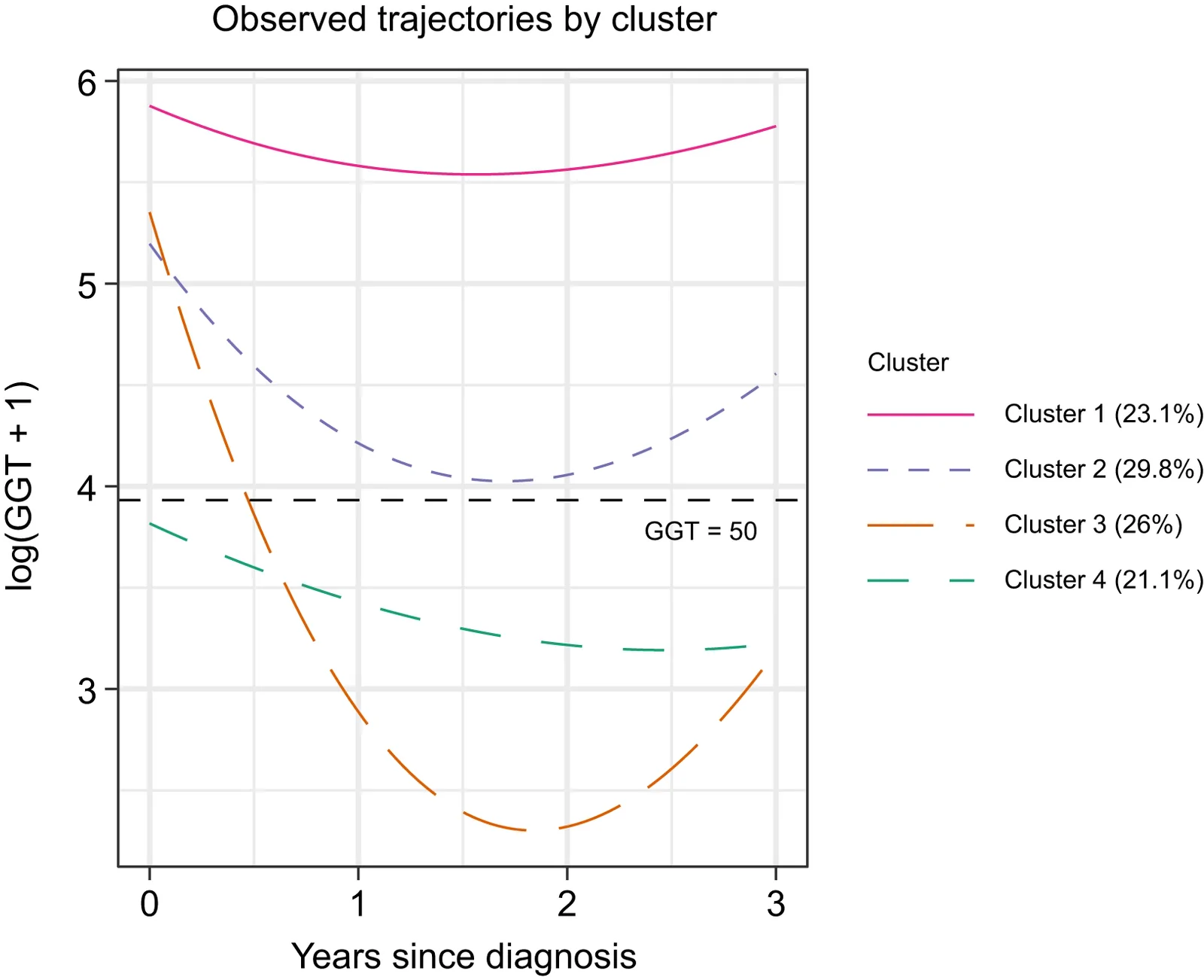
**Observed GGT trajectories by latent class.** Mean log-transformed GGT trajectories [log(GGT + 1)] over 3 yr for four latent classes identified by latent class mixed model analysis. Lines represent LOESS-smoothed trajectories (span = 2) for each cluster. The dashed horizontal line indicates GGT = 50 U/L. **Cluster 1 (pink, solid line, 23.1%): Persistently elevated GGT** remaining >200 U/L throughout follow-up. **Cluster 2 (purple, dashed line, 29.8%): Moderate-stable GGT** with an initial decline but not into the normal range (<50 U/L), followed by a rise towards 90 U/L by Year 3. **Cluster 3 (orange, dashed line, 26.0%): Elevated-declining GGT**, declining steeply well below 50 U/L by Year 1, with a slight rise by Year 3, but remaining <50 U/L. **Cluster 4 (teal, dashed line, 21.1%): Persistently low GGT**, starting below 50 U/L and remaining so throughout follow-up. GGT, gamma-glutamyl transferase; LOESS, locally estimated scatterplot smoothing.

The optimal model, selected based on lowest BIC among models with entropy >0.6, employed log-transformed GGT, four-quantile splines link function, natural cubic splines, and four latent classes (BIC = 6,910, Akaike Information Criterion = 6,770, entropy = 0.628; Fig. 2). Because LCMM assigns membership probabilistically, we also evaluated class separation using posterior membership probabilities. The final four-class model demonstrated good classification certainty with mean posterior class membership probabilities of 0.84 (cluster 3), 0.77 (cluster 4), 0.75 (cluster 1), and 0.6 (cluster 2). The model generated four clusters that each included >20% of the cohort, as well as clinically meaningful trajectory patterns.

We also explored four-cluster models using ALT alone and GGT plus ALT (Fig. S3). The ALT plus GGT model had lower entropy than the ALT-only and GGT-only models. The ALT-only model had one class size that was very small (<10%) and higher BIC than the GGT-only model.

### Characteristics of GGT trajectory clusters

The optimal four-class model identified distinct GGT trajectory patterns (Fig. 2 and Table 2).

**Table 2:** Characteristics by GGT trajectory cluster.

Characteristic	Cluster 1 (n = 181, 23.1%)	Cluster 2 (n = 233, 29.8%)	Cluster 3 (n = 203, 26.0%)	Cluster 4 (n = 165, 21.1%)	*p* value
Trajectory pattern	Persistently elevated	Moderate-stable	Elevated-declining	Persistently low	
Demographics					
Age at diagnosis (yr), median (IQR)	14.6 (11.6–16.5)	13.6 (10.5–16.0)	11.4 (8.0–14.9)	11.8 (8.6–15.0)	<0.001
Male sex, n (%)	131 (72.4)	143 (61.4)	117 (57.6)	85 (51.5)	0.001
Clinical features					
IBD present, n (%)	136 (75.1)	175 (75.1)	172 (84.7)	127 (77.0)	0.059
PSC with AIH features, n (%)	70 (38.7)	87 (37.3)	69 (34.0)	62 (37.6)	0.793
Large duct involvement, n (%)	170 (94.4)	206 (89.6)	169 (84.1)	139 (85.3)	0.008
Medications at baseline					
Ursodeoxycholic acid, n (%)	137 (79.7)	150 (78.9)	122 (84.7)	111 (74.0)	0.160
Corticosteroids, n (%)	66 (43.1)	78 (45.6)	57 (46.0)	63 (48.1)	0.871
Vancomycin, n (%)	32 (21.3)	35 (20.7)	31 (26.3)	24 (18.5)	0.500
Laboratory values at baseline					
GGT (U/L), median (IQR)	401 (254–606)	203 (125–309)	275 (140–486)	36 (17–76)	<0.001
ALT (U/L), median (IQR)	165 (97–254)	109 (60–210)	135 (72–274)	45 (24–122)	<0.001
Haemoglobin (g/dl), mean (SD)	12.2 (2.2)	12.2 (2.2)	12.1 (2.0)	12.5 (1.9)	0.445
Platelets (×10^3^/μl), mean (SD)	321 (156)	302 (151)	366 (147)	313 (131)	<0.001
INR, mean (SD)	1.11 (0.23)	1.15 (0.65)	1.07 (0.14)	1.19 (1.12)	0.387
Albumin (g/dl), mean (SD)	3.90 (0.56)	3.92 (0.61)	4.03 (0.53)	4.12 (0.59)	0.001
Total bilirubin (mg/dl), median (IQR)	0.90 (0.47–1.78)	0.60 (0.40–1.10)	0.50 (0.30–0.87)	0.40 (0.30–0.70)	<0.001
AST (U/L), median (IQR)	126 (78–185)	87 (47–195)	100 (54–190)	44 (28–90)	<0.001
ALP (U/L), median (IQR)	472 (300–702)	340 (206–486)	372 (231–572)	222 (134–326)	<0.001
CRP (mg/dl), median (IQR)	0.80 (0.35–2.20)	0.60 (0.27–1.40)	1.10 (0.50–2.12)	0.40 (0.14–0.67)	0.001
ESR (mm/h), median (IQR)	28 (12–53)	33 (14–60)	39 (19–62)	16 (6–43)	0.002
IgG (mg/dl), mean (SD)	1,565 (968–2,022)	1,460 (958–1,999)	1,556 (1,160–2,059)	1,380 (1,028–1,980)	0.768
Outcomes					
Liver transplant, n (%)	43 (23.8)	28 (12.0)	4 (2.0)	7 (4.2)	<0.001

Note: *p* values from Chi-square tests (categorical), ANOVA (continuous normally distributed), or Kruskal-Wallis test (continuous not normally distributed). AIH, autoimmune hepatitis; ALP, alkaline phosphatase; ALT, alanine aminotransferase; AST, aspartate aminotransferase; CRP, C-reactive protein; ESR, erythrocyte sedimentation rate; GGT, gamma-glutamyl transferase; IBD, inflammatory bowel disease; INR, international normalised ratio; PSC, primary sclerosing cholangitis.

**Cluster 1 (persistently elevated, n = 181, 23.1%):** On average, GGT remained above 200 U/L throughout follow-up (baseline median 401 [IQR 254–606] U/L). This cluster had the highest proportion of males (72.4%) and large duct involvement (94.4%), oldest age at diagnosis (median 14.6 [IQR 11.6–16.5] yr), most abnormal biochemistry (including highest GGT, ALT, ALP, aspartate aminotransferase [AST], and total bilirubin, and lowest albumin), and highest transplant rate (23.8%).

**Cluster 2 (moderate-stable, n = 233, 29.8%):** Baseline median GGT was 203 (IQR 125–309) U/L and, on average, did not decrease below 50 U/L over follow-up. Patients were generally younger than those in cluster 1, but older than those in clusters 3 and 4, with intermediate male prevalence and biochemical abnormalities. The transplant rate (12.0%) was also intermediate between the high rate in cluster 1 and low rates in clusters 3 and 4.

**Cluster 3 (elevated-declining, n = 203, 26.0%):** Baseline median GGT was elevated (275 [IQR 140–486] U/L), on average, but declined rapidly to below 50 U/L by 1 yr, remaining low until the end of follow-up at Year 3. Despite elevated baseline GGT, these patients had very good outcomes (2.0% transplant rate). They were youngest (median 11.4 [IQR 8.0–14.9] yr), had highest prevalence of IBD (84.7%), and highest platelet counts.

**Cluster 4 (persistently low, n = 165, 21.1%):** GGT remained below 50 U/L throughout (baseline median 36 [IQR 17–76] U/L), with mildest biochemical abnormalities and 4.2% transplant rate. This group had the lowest male prevalence (51.5%).

Globally, the four clusters differed significantly in age, sex, large duct involvement, baseline GGT, ALT, platelets, albumin, total bilirubin, AST, ALP, C-reactive protein, erythrocyte sedimentation rate, and transplant rates (all *p* <0.05), with a trend towards significance for IBD (*p* = 0.059). Medication use did not differ across clusters. Median baseline fibrosis stage on biopsy (METAVIR scoring system, F0–F4) was 2 in all four clusters, although the underlying stage distribution differed significantly across clusters (Kruskal-Wallis *p* = 0.004), with a greater proportion of advanced (stage 3–4) disease in cluster 1.

Specific pairwise comparisons of clusters 1 (worst outcome) and 2 (intermediate outcome) *vs*. cluster 3 (good outcome) revealed several clinically important differences (Table S2). Relative to cluster 3, cluster 1 (worst outcome) was characterised by older age, more males, less frequent IBD, more frequent large duct involvement, and more abnormal baseline biochemistry. Relative to cluster 3, cluster 2 (intermediate outcome) was also characterised by older age and less frequent IBD, but not more abnormal baseline biochemistry. In fact, baseline GGT, ALT, and ALP were significantly lower in cluster 2 than in cluster 3, suggesting the prognostic importance of GGT (and biochemistry more broadly) trajectory rather than baseline value alone.

### GGT trajectories and transplant-free survival

**Fig. 3:**
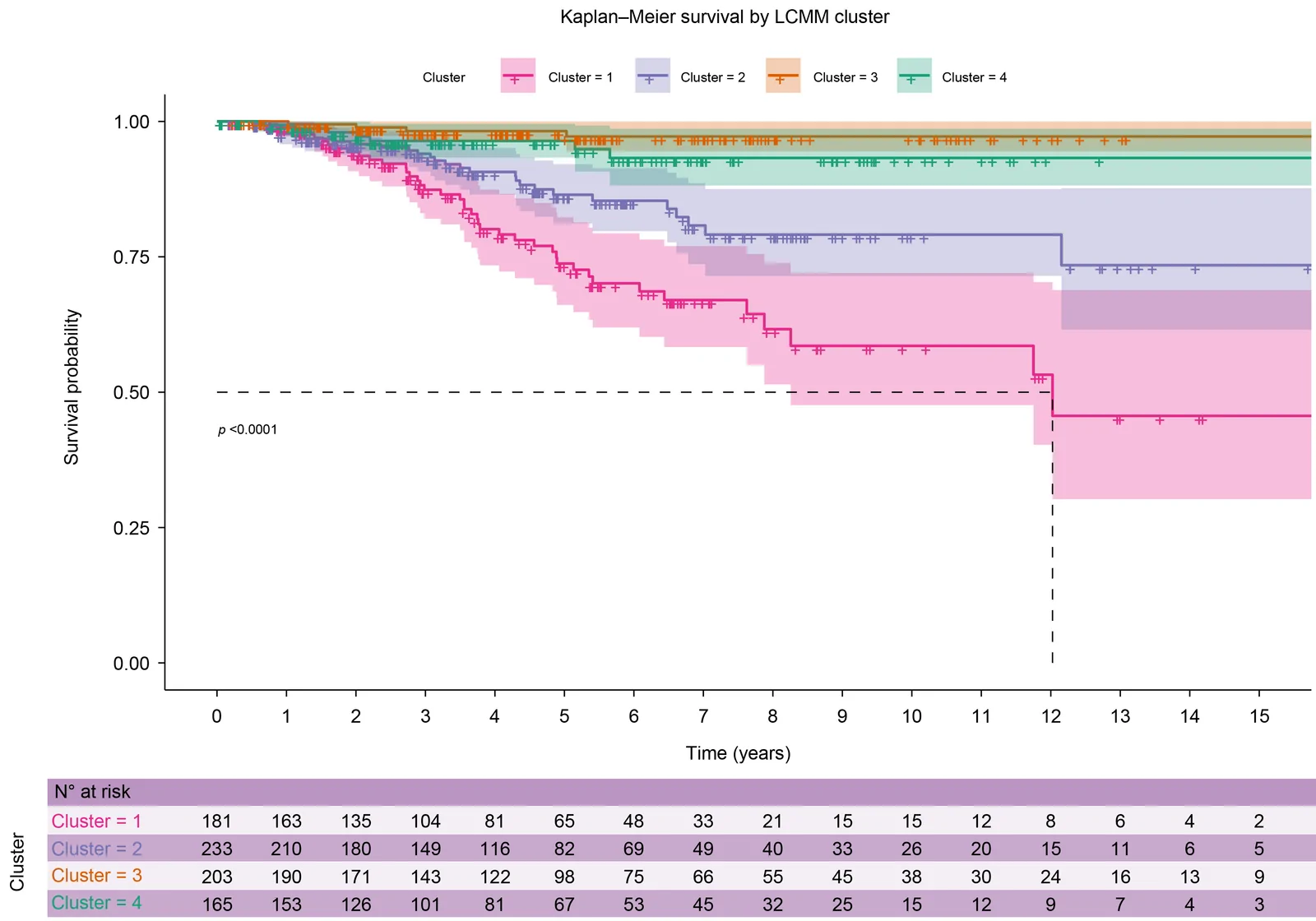
**Kaplan-Meier transplant-free survival by GGT trajectory cluster.** Kaplan-Meier curves showing transplant-free survival over 15 yr of follow-up, stratified by GGT trajectory cluster. Shaded regions indicate 95% CIs. Risk table below the plot shows number at risk at yearly intervals. Log-rank test *p* <0.0001.**5-yr transplant-free survival estimates:** Cluster 1 (persistently elevated): 74%; cluster 2 (moderate-stable): 86%; cluster 3 (elevated-declining): 98%; cluster 4 (persistently low): 96%. The curves demonstrate clear separation, with cluster 1 having substantially worse outcomes than all other clusters. Clusters 3 and 4 have similar favourable outcomes, whereas cluster 2 has an intermediate outcome. GGT, gamma-glutamyl transferase; LCMM, latent class mixed modelling.

Kaplan-Meier analysis revealed important differences in transplant-free survival across the four GGT trajectory clusters (log-rank *p* <0.0001; Fig. 3). The 5-yr transplant-free survival estimates were 74% for cluster 1 (persistently elevated), 86% for cluster 2 (moderate-stable), 98% for cluster 3 (elevated-declining), and 96% for cluster 4 (persistently low).

In multivariable Cox proportional hazards analysis adjusted for age, sex, IBD status, PSC with AIH features, large duct involvement, and medications, GGT trajectory cluster remained the strongest predictor of outcomes (Table 3). Compared with cluster 4 (persistently low GGT), cluster 1 (persistently elevated) had 6.4-fold increased hazard of transplant (HR 6.45, 95% CI 2.87–14.5, *p* <0.001). Cluster 2 (moderate-stable) had threefold increased hazard (HR 3.00, 95% CI 1.31–6.90, *p* = 0.010). The risk of progression to transplant in cluster 3 (elevated-declining) was similar to that in cluster 4 (persistently low) (HR 0.46, 95% CI 0.13–1.60, *p* = 0.22). The model demonstrated good discrimination (concordance index 0.758) and satisfied proportional hazards assumptions (global test *p* = 0.15).

**Table 3:** Multivariable Cox proportional hazards model for transplant-free survival.

Variable	Hazard ratio	95% CI	*p* value
**GGT trajectory cluster (ref: cluster 4 - persistently low)**			
Cluster 1 (persistently elevated)	6.45	2.87–14.5	<0.001
Cluster 2 (moderate-stable)	3.00	1.31–6.90	0.010
Cluster 3 (elevated-declining)	0.46	0.13–1.60	0.22
Age at diagnosis, per year	1.00	0.94–1.05	0.85
Male sex	0.79	0.50–1.23	0.30
IBD present	0.75	0.45–1.25	0.27
PSC with AIH features	0.76	0.47–1.23	0.27
Large duct involvement	2.07	0.65–6.65	0.22
Ursodeoxycholic acid	0.67	0.35–1.27	0.22
Corticosteroids	0.76	0.48–1.19	0.23
Vancomycin	2.00	1.29–3.10	0.002

Note: that *p* values for individual covariates are from the multivariable Cox proportional hazards model (Wald test). Model statistics: n = 774 patients, 82 events; concordance index = 0.758; likelihood ratio test: χ^2^ = 79.67 on 11 df, *p* <0.001; proportional hazards assumption satisfied (global *p* = 0.15). AIH, autoimmune hepatitis; GGT, gamma-glutamyl transferase; IBD, inflammatory bowel disease; PSC, primary sclerosing cholangitis.

### Sensitivity analyses

In a sensitivity analysis using complete-case deletion for patients with any missing covariate data, including exclusion of patients without medication start dates (n = 549, 68 events), results were qualitatively similar to the primary analysis (Table S3). GGT trajectory cluster remained the strongest predictor, with cluster 1 showing HR 6.03 (95% CI 2.51–14.5, *p* <0.001) and cluster 2 showing HR 2.78 (95% CI 1.11–6.92, *p* = 0.028) compared with cluster 4. Concordance was 0.728 *vs*. 0.758.

A sensitivity analysis restricted to patients with high posterior class membership probability (>0.80) confirmed consistent associations for the elevated-declining (cluster 3) and persistently elevated (cluster 1) phenotypes, though the model was underpowered for the moderate-stable group (cluster 2) (Table S3).

### Prediction of cluster membership

To assess whether cluster membership could be predicted using baseline and early follow-up data, we used a traditional multinomial logistic regression framework. Using only GGT values at baseline and 6 months (log-transformed), the model achieved excellent discrimination for identifying cluster membership, with multiclass AUC of 0.835 and per-class AUCs ranging from 0.77 to 0.86 (Table S4). Adding baseline demographic and clinical variables (age, sex, IBD status, PSC with AIH features, large duct involvement) did not significantly improve performance (multiclass AUC of 0.84). Addition of comprehensive baseline laboratory values did not substantially improve prediction either (multiclass AUC 0.813), suggesting that GGT measurements at two early time points are the primary drivers of cluster assignment. Addition of ALT did not improve predictive performance (multiclass AUC 0.831).

In the parsimonious model using only baseline and 6-month log(GGT) values, high baseline log(GGT) strongly predicted membership in all three non-reference clusters relative to cluster 4 (persistently low), with odds ratios (ORs) of 11.0 (95% CI 6.6–18.5) for cluster 1 (persistently elevated), 6.4 (95% CI 4.2–9.7) for cluster 2 (moderate-stable), and 14.3 (95% CI 8.8–23.2) for cluster 3 (elevated-declining). In addition, the 6-month log(GGT) value differentiated high-risk from low-risk trajectories among patients with elevated baseline GGT. Specifically, a higher 6-month GGT predicted cluster 1 membership (OR 3.2, 95% CI 1.8–5.9), whereas a lower 6-month GGT strongly predicted cluster 3 membership (OR 0.16, 95% CI 0.09–0.29), with cluster 2 showing no significant change (OR 0.84, 95% CI 0.50–1.40). These findings suggest that patients with elevated GGT at diagnosis who fail to show decline by 6 months are at substantially higher risk for adverse outcomes.

## Discussion

In this large multicentre study of 782 paediatric PSC patients, we applied LCMM to serial GGT measurements and identified four distinct trajectory phenotypes with different prognostic implications. Our study represents the first application of trajectory modelling to PSC. Our findings demonstrate that longitudinal GGT patterns over the first 3 yr after PSC diagnosis capture clinically meaningful disease heterogeneity beyond what single time point assessments can provide, with trajectory cluster membership strongly predicting transplant-free survival.

Conventional prognostic models in PSC, including the Mayo PSC Risk Score, Primary Sclerosing Cholangitis Risk Estimate Tool (PREsTo), and the paediatric SCOPE Index, rely on cross-sectional variables measured at a single time point.^5^^,^^11^^,^^12^ These do not capture how biochemical profiles evolve over time or distinguish patients who follow different longitudinal patterns from a similar starting point. Notably, although static GGT is included in the SCOPE Index, its contribution to model discrimination is marginal compared with bilirubin and albumin, suggesting that GGT's prognostic value lies in its dynamic trajectory over time rather than in its absolute level. LCMM addresses this limitation by identifying subgroups following similar longitudinal patterns,^6^ an approach successfully applied in other chronic diseases.^7^^,^^8^^,^^13^

The four trajectory clusters have distinct clinical implications. Patients with persistently elevated GGT (cluster 1) experienced the worst outcomes, whereas those with persistently low GGT (cluster 4) had excellent prognosis, as expected. Most informative was the comparison between clusters 2 and 3, which started with similarly elevated baseline GGT but diverged thereafter. Cluster 3 achieved rapid GGT normalisation and transplant-free survival comparable with cluster 4, whereas cluster 2 showed only partial decline and experienced significantly worse outcomes. This underscores that baseline GGT alone inadequately captures prognosis. The trajectory of change, particularly within the first 6–12 months, provides critical information. The fact that rapid GGT normalisers achieved outcomes comparable with those with persistently normal GGT suggests that achieving normalisation confers a similar favourable prognosis regardless of baseline values.

The prognostic importance of early biochemical response aligns with prior Consortium work demonstrating that patients with GGT <50 U/L at 1 yr had a 5-yr event-free survival of 91% compared with 67% in non-normalisers.^4^ Along the same lines, in a single-centre Dutch cohort of 43 paediatric PSC patients, GGT decrease >25% within 1 yr predicted favourable 5-yr outcomes.^14^ However, both studies used binary GGT classifications at discrete time points. Our trajectory-based approach moves beyond this dichotomy by identifying four distinct longitudinal phenotypes, including an intermediate group (cluster 2) with persistently moderately elevated GGT whose outcomes fall between the extremes, a subgroup that binary classification would miss.

About one-third of paediatric PSC patients display overlapping features of AIH,^2^ which is characterised by higher liver enzymes such as ALT. Whether this constitutes a separate disease entity or simply a more inflammatory phenotype of PSC that predominates in children is debatable.^15^ Notably, ALT, reflective of hepatocellular inflammation and the AIH overlap phenotype, did not identify prognostically meaningful trajectory clusters when modelled alone, nor did it enhance discrimination when added to GGT. Importantly, this should not be interpreted as evidence that hepatocellular inflammation is of lesser intrinsic biological importance. Hepatocellular inflammation, particularly in the AIH overlap phenotype, is often effectively suppressed by corticosteroids and other immunosuppressive therapies, which may attenuate its observable impact on long-term outcome. Conversely, no equally effective therapy exists for the biliary component of PSC. The relative prognostic dominance of GGT trajectory in our analysis may therefore reflect, at least in part, an asymmetry in the effectiveness of available therapies rather than a difference in the underlying pathogenic importance of the two compartments.

Baseline characteristics revealed consistent patterns across trajectory groups. Patients in unfavourable clusters (1 and 2) were older at diagnosis, more often male, had higher rates of large duct involvement, lower albumin, and higher liver enzymes. Conversely, favourable trajectory clusters (3 and 4) were characterised by younger age, higher platelet counts and albumin, and higher prevalence of IBD. The favourable association of concomitant IBD with GGT trajectory we observed contrasts with adult literature, in which IBD has been linked to worse hepatobiliary outcomes. Leung *et al.*^15^ recently reported higher mortality in adults with PSC-IBD compared with PSC alone, with diagnostic sequence also influencing outcomes. In children, however, PSC-IBD is overwhelmingly diagnosed through routine liver biochemistry surveillance in established IBD, identifying PSC at an earlier and less severe stage. An earlier analysis of this Consortium cohort demonstrated that children with PSC-IBD present with milder baseline disease (lower Model for End-Stage Liver Disease [MELD] score, AST-to-Platelet Ratio Index, ALT) than those without IBD, and that adjustment for baseline severity eliminates the apparent protective effect of IBD on event-free survival (univariate HR 0.66, attenuated to MELD-adjusted HR 0.88, *p* = 0.58), supporting lead-time bias as the predominant explanation.^16^ Younger age at diagnosis was associated with favourable clusters, increasing the possibility of age-related lead-time bias. However, trajectory cluster membership remained independently associated with transplant-free survival after adjusting for age at diagnosis in multivariable Cox regression, suggesting that GGT trajectory captures prognostic information beyond what age alone explains. Baseline medication exposure did not differ across clusters, suggesting trajectory patterns reflect disease biology rather than treatment effects.

Notably, the increased hazard associated with vancomycin exposure in Table 3 should not be interpreted as a sign of harm, as it may well reflect confounding by indication. Oral vancomycin is preferentially used in children with refractory PSC, persistent GGT elevation, or active IBD. The present analysis was not designed to estimate the causal effect of vancomycin in paediatric PSC. Rigorous inference would require a propensity score matching or comparable causal approach.^17^

The ability to predict trajectory cluster membership using baseline and 6-month GGT values has practical implications. Patients with elevated baseline GGT who fail to improve by 6 months can be identified as high risk, warranting closer surveillance or earlier intervention. These findings also have important implications for clinical trial design in PSC, where demonstrating treatment efficacy has been hindered by clinical heterogeneity as well as the rarity of and time to achieve difficult endpoints.^18^ Trajectory-based phenotyping could enable enrichment strategies selecting patients most likely to progress, allowing trials to achieve adequate power with smaller sample sizes. Furthermore, GGT trajectory may serve as an early surrogate endpoint; a treatment that shifts patients from an unfavourable to a favourable trajectory within 6–12 months would provide a much earlier signal of efficacy than waiting years for transplant data.

This study has several strengths, including large sample size, systematic evaluation of 36 candidate models with objective selection criteria, and novel approach to examining serial GGT in paediatric PSC. Limitations include the retrospective design with potential selection bias, GGT measurements from routine care rather than standardised protocols, inability to exclude treatment effects on trajectories, and need for external validation. In performing a sensitivity analysis to explore potential classification uncertainty, associations remained consistent for the elevated-declining (cluster 3) and persistently elevated (cluster 1) phenotypes, but the examination was underpowered to evaluate the moderate-stable (cluster 2) group. The mean posterior probability for cluster 2 was 0.6 (*vs*. >0.75 for the others), suggesting this subgroup may represent a continuum between the high and low extremes rather than a discrete phenotype.

The decline in GGT availability over follow-up merits discussion. The dominant driver of attrition was transition to adult care, which at most participating centres occurs at a defined age largely independent of clinical status. Patients lost to follow-up should therefore not differ markedly from those retained, although some exits for clinical reasons cannot be excluded. However, every patient contributed a baseline GGT measurement and 81% had a value at 1 yr, so cluster assignment drew on substantial early data. LCMM further accommodates incomplete follow-up by using all available measurements under a missing-at-random assumption. These features limit, though do not eliminate, the potential for attrition to bias trajectory assignment. Prospective cohorts with mandated longitudinal sampling will be needed to confirm trajectory phenotype prevalence beyond the first 3 yr.

In conclusion, LCMM identifies four distinct GGT trajectory phenotypes in paediatric PSC with strong prognostic implications. Early trajectory patterns, particularly GGT response within the first 6–12 months, differentiate patients with similar baseline values but divergent outcomes. These findings support incorporation of longitudinal biochemical assessment into prognostic evaluation and suggest that trajectory-based phenotyping could enhance clinical trial design.

## Abbreviations

AIH, autoimmune hepatitis; ALP, alkaline phosphatase; ALT, alanine aminotransferase; AST, aspartate aminotransferase; BIC, Bayesian Information Criterion; CRP, C-reactive protein; ESR, erythrocyte sedimentation rate; GGT, gamma-glutamyl transferase; HR, hazard ratio; IBD, inflammatory bowel disease; INR, international normalised ratio; LCMM, latent class mixed modelling; LOESS, locally estimated scatterplot smoothing; MELD, Model for End-Stage Liver Disease; OR, odds ratio; PSC, primary sclerosing cholangitis; SCOPE, Sclerosing Cholangitis Outcomes in Pediatrics; SMD, standardised mean difference; UDCA, ursodeoxycholic acid.

## Authors’ contributions

Conceptualization, methodology, investigation, data curation, formal analysis, writing – original draft, supervision, project administration: AR. Methodology, formal analysis, supervision: KL. Conceptualization, resources, data curation, supervision, writing – review and editing: MD. Data curation, formal analysis, writing – original draft: DD.

Software, formal analysis, visualization: YEP. Investigation, resources, writing – review and editing: M. Amin, M. Aumar, M. Auth, EDTF, WEM, KNF, NG, SG, SH, MKJ, BMK, BGPK, KML, CM, MM, NO, AP, ERP, PS, PV, ARF, JPS, MET.

## Data availability

Data are available from the Pediatric PSC Consortium upon reasonable request to the corresponding authors.

## Declaration of generative AI and AI-assisted technologies in the manuscript preparation process

During the preparation of this work, the authors used Claude (Anthropic) to assist with condensing and restructuring portions of the Introduction and Discussion. After using this tool, the authors reviewed and edited the content as needed and take full responsibility for the content of the published article.

## Financial support

No financial support was received to produce this manuscript.

## Conflicts of interest

The authors declare no conflicts of interest that pertain to this work.

Please refer to the accompanying ICMJE disclosure forms for further details.
