# Supplementary material for: Longitudinal GGT trajectories identify prognostic phenotypes in paediatric primary sclerosing cholangitis
Source: JHEP Rep. 2026 Jun 26;8(10):101940. doi: 10.1016/j.jhepr.2026.101940 (PMC13545721; doi:10.1016/j.jhepr.2026.101940)
Supplement: Supplementary file 1 [file mmc1.pdf]

Longitudinal GGT trajectories identify prognostic phenotypes in paediatric primary sclerosing cholangitis

Daniela Denier, Yiming (Emmett) Peng, Mansi Amin, Madeleine Aumar, Marcus Auth, Eleonora Druve Tavares Fagundes, Wael El-Matary, Alexandre R. Ferreira, Katryn N. Furuya, Nitika Gupta, Stephen Guthery, Simon Horslen, M. Kyle Jensen, Binita M. Kamath, Bart G.P. Koot, Kathleen M. Loomes, Cara Mack, Mercedes Martinez, Nadia Ovchinsky, Alexandra Papadopoulou, Emily R. Perito, Pushpa Sathya, James P. Stevens, M. Elizabeth Tessier, Pamela Valentino, Mark Deneau, Kuan Liu, Amanda Ricciuto

Table of contents

Fig. S1……2
Fig. S2……3
Fig. S3……4
Table S1……5
Table S2……6
Table S3……7
Table S4……8

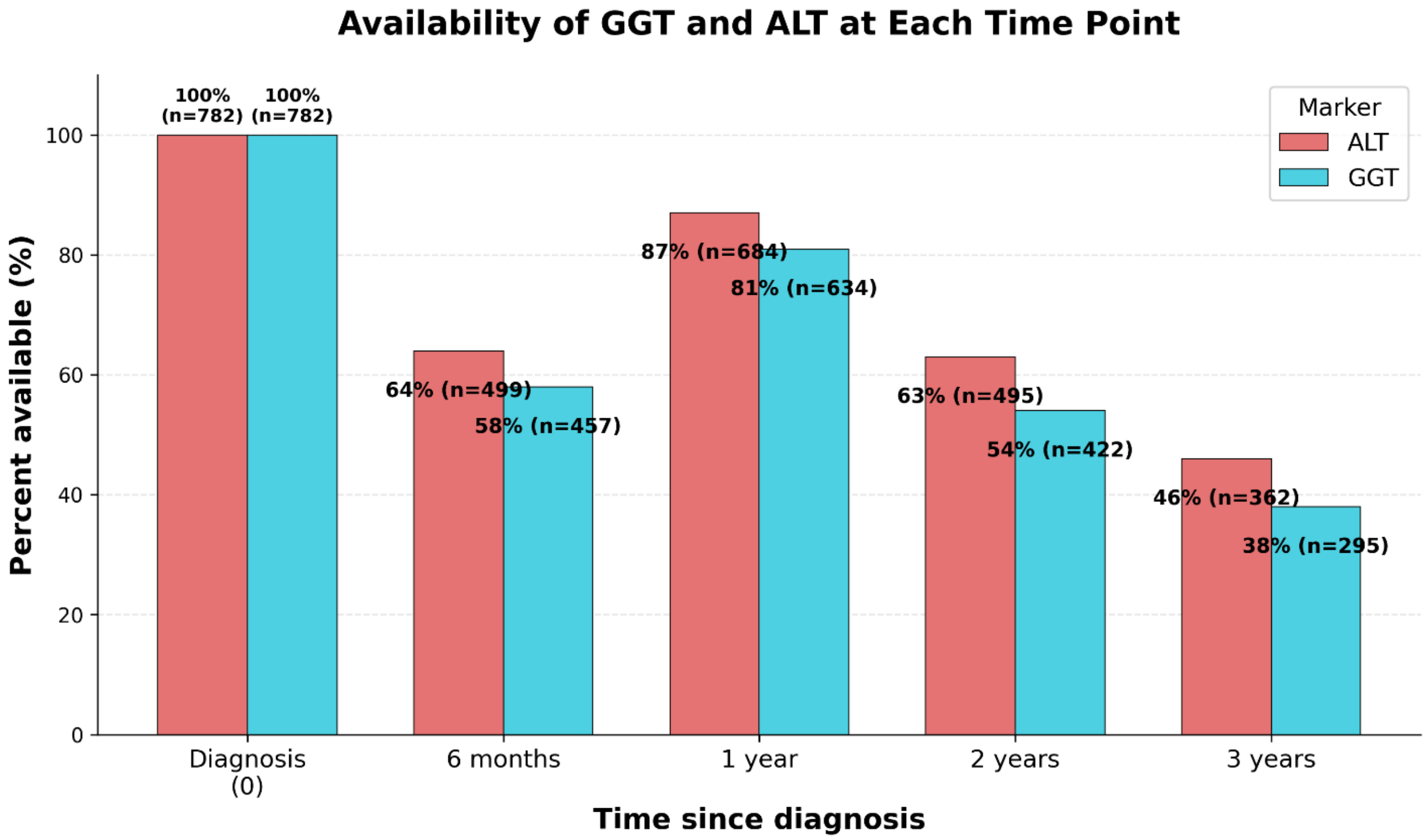

Fig. S1. Availability of GGT and ALT Measurements Over Time

Bar chart showing percentage of patients with available GGT (teal) and ALT (pink) measurements at each time point (baseline, 6 months, 1 year, 2 years, 3 years). GGT: 100% at baseline, 58% at 6 months, 81% at 1 year, 54% at 2 years, and 38% at 3 years. ALT availability: 100% at baseline, 64% at 6 months, 87% at 1 year, 63% at 2 years, and 46% at 3 years.

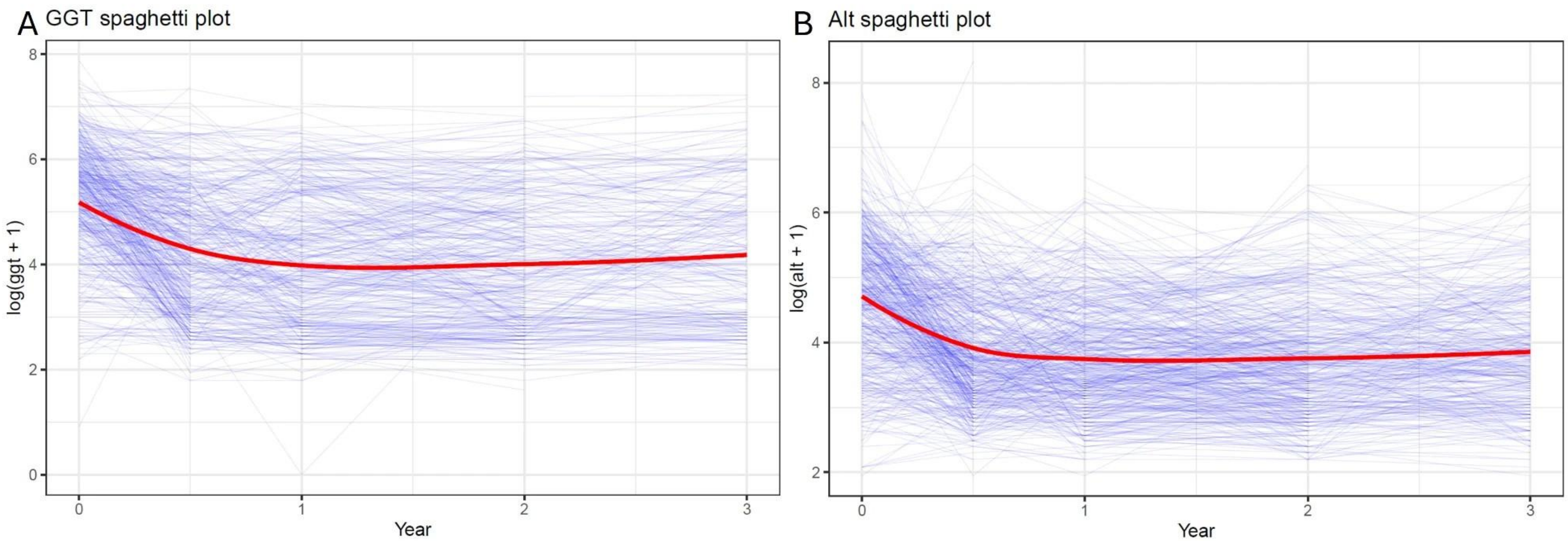

Fig. S2. Individual Patient GGT and ALT Trajectories (Spaghetti Plot)

Spaghetti plots showing all individual patient trajectories (n=782) as semi-transparent blue lines, with red LOESS-smoothed overall trend lines. Panel A shows GGT trajectories and Panel B shows ALT trajectories, both plotted versus years since diagnosis (0-3 years).
These visualisations demonstrate the heterogeneity in individual lab trajectories underlying the latent class solution. The overall trend lines (red) show modest average decline for both markers, masking the clinically important heterogeneity underlying.

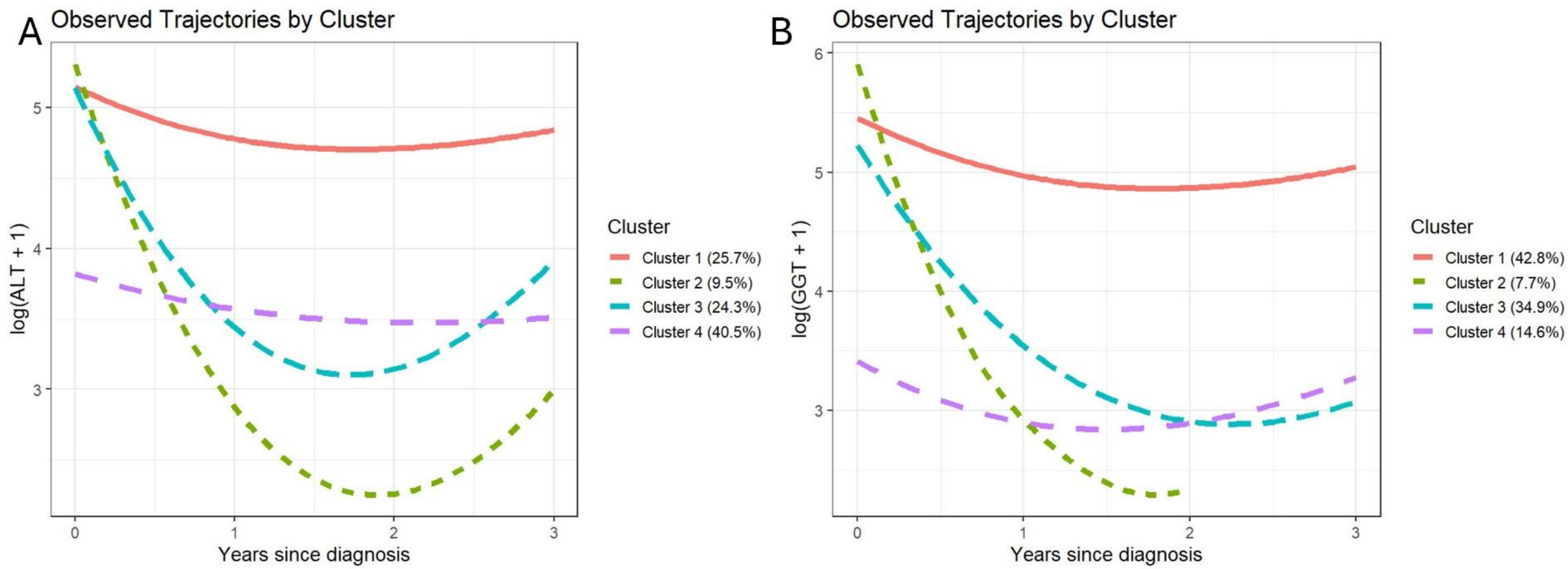

Fig. S3. 4-Cluster Models with ALT and GGT Plus ALT

(A) Latent class mixed model trajectories using ALT measurements alone identified four clusters. **(B)** Combined ALT and GGT trajectory modelling also identified four clusters. Y-axis shows log-transformed values.

Table S1. Model Selection Results for Latent Class Mixed Models

Rank	Transform	Link	Shape	Classes	BIC	AIC	Entropy
**1***	Log	4-quant splines	Splines (df=3)	4	6910.1	6770.2	0.628
**2**	Log	4-quant splines	Quadratic	3	7092.7	6994.8	0.651
**3**	Log	4-quant splines	Linear	3	7576.3	7492.4	0.762
**4**	Log	Linear	Linear	4	7839.4	7755.5	0.818
**5**	Raw	4-quant splines	Splines (df=3)	4	29854.8	29714.9	0.666
**6**	Raw	4-quant splines	Quadratic	3	30027.9	29930.0	0.659
**7**	Raw	4-quant splines	Quadratic	4	30047.7	29926.4	0.774
**8**	Raw	4-quant splines	Linear	3	30508.2	30424.3	0.762
**9**	Raw	4-quant splines	Linear	4	30524.9	30422.3	0.810
**10**	Raw	Linear	Linear	4	34297.0	34213.1	0.736

Abbreviations: AIC, Akaike information criterion; BIC, Bayesian information criterion; df, degrees of freedom; quant, quantile.

Note: *Selected model (Rank 1): Log transformation, 4-quantile splines link, natural cubic splines trajectory, 4 classes. Selection criteria: Lowest BIC among models with entropy >0.6 (good class separation); 4-class solution provides clinically meaningful patterns; Entropy of 0.628 indicates acceptable classification certainty. Only top 10 of 36 models shown.

Table S2. Key Pairwise Comparisons Between GGT Trajectory Clusters

A. Cluster 1 (Persistently Elevated) vs Cluster 3 (Elevated-Declining)

Characteristic	Cluster 1 (n=181)	Cluster 3 (n=203)	p-value
**Age at diagnosis, years, median (IQR)**	14.6 (11.6-16.5)	11.4 (8.0-14.9)	<0.001
**Male sex, n (%)**	131 (72.4)	117 (57.6)	0.004
**IBD present, n (%)**	136 (75.1)	172 (84.7)	0.026
**Large duct involvement, n (%)**	170 (94.4%)	169 (84.1%)	0.002
**Baseline GGT, U/L, median (IQR)**	401 (254-606)	275 (140-486)	<0.001
**Platelets, ×10³/μL, mean (SD)**	321 (156)	366 (147)	0.004
**Albumin, g/dL, mean (SD)**	3.90 (0.56)	4.03 (0.53)	0.023
**AST, U/L, median (IQR)**	126 (78-185)	100 (54-190)	0.036
**ALP, U/L, median (IQR)**	472 (300-702)	372 (231-572)	0.008
**Total bilirubin, mg/dL, median (IQR)**	0.90 (0.47-1.78)	0.50 (0.30-0.87)	<0.001
**Transplant, n (%)**	43 (23.8)	4 (2.0)	<0.001

B. Cluster 2 (Moderate-Stable) vs Cluster 3 (Elevated-Declining)

Characteristic	Cluster 2 (n=233)	Cluster 3 (n=203)	p-value
**Age at diagnosis, years, median (IQR)**	13.6 (10.5-16.0)	11.4 (8.0-14.9)	<0.001
**IBD present, n (%)**	175 (75.1)	172 (84.7)	0.018
**Baseline GGT, U/L, median (IQR)**	203 (125-309)	275 (140-486)	<0.001
**Baseline ALT, U/L, median (IQR)**	109 (60-210)	135 (72-274)	0.048
**Platelets, ×10³/μL, mean (SD)**	302 (151)	366 (147)	<0.001
**Total bilirubin, mg/dL, median (IQR)**	0.60 (0.40-1.10)	0.50 (0.30-0.87)	0.003
**Alkaline phosphatase, U/L, median (IQR)**	340 (206-486)	372 (231-572)	0.035
**Transplant, n (%)**	28 (12.0)	4 (2.0)	<0.001

Note: P-values from chi-square tests (categorical variables), t-tests (continuous normally distributed variables) or Mann-Whitney U tests (continuous not normally distributed variables).

Table S3. Sensitivity Analyses: Cox Proportional Hazards Regression for Transplant-Free Survival

	Sensitivity Analysis 1: Complete Case (n=549)			Sensitivity Analysis 2: Posterior Probability >0.8 (n=342)		
	**HR**	**95% CI**	**p-value**	**HR**	**95% CI**	**p-value**
**GGT trajectory cluster (ref: Cluster 4 - persistently low)**						
**Cluster 1 (persistently elevated)**	6.03	2.51–14.5	<0.001	20.0	2.64–151	0.004
**Cluster 2 (moderate-stable)**	2.78	1.11–6.92	0.028	6.39	0.65–63.0	0.11
**Cluster 3 (elevated-declining)**	0.53	0.13–2.13	0.37	2.18	0.23–21.2	0.50
**Age at diagnosis, per year**	1.00	0.94–1.06	>0.9	1.05	0.96–1.16	0.30
**Male sex**	0.77	0.47–1.27	0.31	0.57	0.26–1.23	0.15
**IBD present**	0.83	0.47–1.48	0.53	1.02	0.39–2.66	>0.9
**PSC with AIH features**	0.90	0.53–1.54	0.70	1.04	0.44–2.43	>0.9
**Large duct involvement**	1.63	0.50–5.28	0.42	1.33	0.29–6.18	0.71
**Ursodeoxycholic acid**	0.47	0.17–1.31	0.15	1.03	0.29–3.69	>0.9
**Corticosteroids**	0.73	0.43–1.24	0.25	0.80	0.34–1.91	0.62
**Vancomycin**	1.39	0.72–2.68	0.33	1.15	0.45–2.95	0.78

Abbreviations: AIH, autoimmune hepatitis; CI, confidence interval; GGT, gamma-glutamyl transferase; HR, hazard ratio; IBD, inflammatory bowel disease; PSC, primary sclerosing cholangitis.

Sensitivity Analysis 1 model statistics: n = 549 patients, 68 events; Concordance index = 0.728; Likelihood ratio test: $\chi^2 = 50.42$ on 11 df, $p < 0.001$; Proportional hazards assumption satisfied (global test p = 0.61).

Sensitivity Analysis 2 model statistics: n = 259 patients (342 met posterior probability threshold; 83 excluded due to missing covariates), 30 events; Concordance index = 0.789; Likelihood ratio test: $\chi^2 = 37.43$ on 11 df, $p < 0.001$.

Comparison to primary analysis: Sensitivity Analysis 1 uses complete-case deletion, excluding patients with any missing covariate data. Results are qualitatively similar to the primary results, with GGT trajectory cluster remaining the strongest predictor of outcomes. Sensitivity Analysis 2 restricted the cohort to patients with posterior class membership probability >0.8, retaining 342 of 782 patients (43.7%). Despite substantially reduced sample size and wider confidence intervals, the direction and magnitude of cluster effects were consistent with the primary analysis, with Cluster 1 showing a markedly elevated hazard ratio (HR 20.0), likely amplified by the exclusion of patients with uncertain classification near cluster boundaries. The loss of statistical significance for Cluster 2 (p = 0.11) and all non-cluster covariates likely reflects the reduced power from only 30 events.

Note: P-values for individual covariates are from the Cox proportional hazards models (Wald test).

Table S4. Performance of Multinomial Logistic Regression Models for Predicting Cluster Membership

Model	Predictors	Multiclass AUC	Cluster 1 AUC	Cluster 2 AUC	Cluster 3 AUC	Cluster 4 AUC
**1**	GGT (baseline, 6m) log-transformed	0.835	0.834	0.768	0.860	0.859
**2**	GGT + demographics + clinical	0.838	0.831	0.760	0.878	0.865
**3**	GGT + demographics + clinical + comprehensive labs	0.813	0.831	0.699	0.852	0.850
**4**	GGT + ALT (baseline, 6m)	0.831	0.829	0.751	0.869	0.856

Abbreviations: AIH, autoimmune hepatitis; ALP, alkaline phosphatase; ALT, alanine aminotransferase; AST, aspartate aminotransferase; AUC, area under receiver operating characteristic curve; CRP, C-reactive protein; ESR, erythrocyte sedimentation rate; GGT, gamma-glutamyl transferase; IBD, inflammatory bowel disease; IgG, immunoglobulin G; INR, international normalised ratio; PSC, primary sclerosing cholangitis.

Model descriptions:

Model 1: log(GGT) at baseline and 6 months only
Model 2: Model 1 + age, sex, IBD, PSC with AIH features, large duct involvement
Model 3: Model 2 + haemoglobin, platelets, INR, sodium, creatinine, protein, albumin, bilirubin, AST, ALP, CRP, ESR, IgG
Model 4: log(GGT) and log(ALT) at baseline and 6 months

Interpretation: Model using only GGT at two early timepoints achieved excellent discrimination (AUC >0.83). Adding clinical variables or additional laboratory data did not substantially improve prediction. ALT measurements provided minimal additional value beyond GGT.

Note: All models developed using multinomial logistic regression fitted on a 70% training set and evaluated on a 30% held-out test set. Missing data imputed with median (continuous) or mode (categorical) values. Multiclass AUC represents overall discrimination across all four clusters.
